# Supplementary material for: Heavy metal distribution and ecological risk in surface sediments of the Bohai Sea
Source: PLoS One. 2025 Jun 27;20(6):e0326701. doi: 10.1371/journal.pone.0326701 (PMC12204586; doi:10.1371/journal.pone.0326701)
Supplement: S1 Table — (DOCX) [file pone.0326701.s001.docx]

| **S1 Table. Geographical information of sampling stations.** | | |
| --- | --- | --- |
| **Sampling station** | **East longitude** | **North latitude** |
| 1 | 119.38 | 39.01 |
| 2 | 119.59 | 39.00 |
| 3 | 119.80 | 39.00 |
| 4 | 119.99 | 39.00 |
| 5 | 120.20 | 39.00 |
| 6 | 120.38 | 39.00 |
| 7 | 120.60 | 39.00 |
| 8 | 119.18 | 38.79 |
| 9 | 119.38 | 38.79 |
| 10 | 119.59 | 38.79 |
| 11 | 119.79 | 38.78 |
| 12 | 120.19 | 38.79 |
| 13 | 120.38 | 38.79 |
| 14 | 120.59 | 38.78 |
| 15 | 119.18 | 38.59 |
| 16 | 119.59 | 38.59 |
| 17 | 119.79 | 38.60 |
| 18 | 119.99 | 38.59 |
| 19 | 120.21 | 38.59 |
| 20 | 120.39 | 38.59 |
| 21 | 120.59 | 38.59 |
| 22 | 119.19 | 38.41 |
| 23 | 119.60 | 38.39 |
| 24 | 119.79 | 38.39 |
| 25 | 119.99 | 38.39 |
| 26 | 120.20 | 38.39 |
| 27 | 120.39 | 38.39 |
| 28 | 120.58 | 38.40 |
| 29 | 119.38 | 38.20 |
| 30 | 119.60 | 38.20 |
| 31 | 119.79 | 38.20 |
| 32 | 120.00 | 38.20 |
| 33 | 120.21 | 38.20 |
| 34 | 120.39 | 38.20 |
| 35 | 120.58 | 38.20 |
| 36 | 120.83 | 38.21 |
| 37 | 119.38 | 39.01 |
| 38 | 119.59 | 39.00 |
| 39 | 119.80 | 39.00 |
| 40 | 119.99 | 39.00 |
| 41 | 120.20 | 39.00 |
| Penglai 19-3 | 120.08 | 38.27 |
