# Supplementary material for: Heavy metal distribution and ecological risk in surface sediments of the Bohai Sea
Source: PLoS One. 2025 Jun 27;20(6):e0326701. doi: 10.1371/journal.pone.0326701 (PMC12204586; doi:10.1371/journal.pone.0326701)
Supplement: S2 Table — (DOCX) [file pone.0326701.s002.docx]

| **Month** |  | **Cu(****mg/kg)** | **Zn(mg/kg)** | **Pb(mg/kg)** | **Cd(mg/kg)** | **Hg(mg/kg)** | **As(mg/kg)** |
| --- | --- | --- | --- | --- | --- | --- | --- |
| May | Min | 2.990 | 15.840 | 0.700 | 0.080 | 0.001 | 0.010 |
|  | Max | 106.990 | 46.540 | 76.760 | 4.980 | 0.017 | 0.460 |
|  | Avg | 17.490 | 31.290 | 12.860 | 0.580 | 0.005 | 0.230 |
|  | SD | 17.268 | 8.210 | 13.217 | 1.236 | 0.003 | 0.124 |
| Aug | Min | 6.760 | 28.770 | 8.790 | 0.060 | 0.017 | 2.080 |
|  | Max | 34.640 | 61.190 | 32.290 | 0.220 | 0.340 | 13.990 |
|  | Avg | 20.270 | 46.550 | 18.080 | 0.150 | 0.066 | 6.520 |
|  | SD | 7.219 | 7.389 | 5.162 | 0.045 | 0.072 | 2.493 |
| October | Min | 5.270 | 8.610 | 8.220 | 0.090 | 0.003 | 0.040 |
|  | Max | 30.080 | 47.920 | 27.900 | 0.560 | 0.034 | 6.900 |
|  | Avg | 12.980 | 25.520 | 17.500 | 0.160 | 0.008 | 1.930 |
|  | SD | 5.601 | 11.429 | 5.233 | 0.082 | 0.007 | 1.661 |
| December | Min | 2.170 | 5.920 | 0.680 | 0.020 | 0.013 | 0.100 |
|  | Max | 25.750 | 55.800 | 22.600 | 0.270 | 0.129 | 5.730 |
|  | Avg | 13.760 | 28.420 | 12.870 | 0.140 | 0.034 | 2.090 |
|  | SD | 7.747 | 12.304 | 6.539 | 0.071 | 0.020 | 1.432 |

**S2 Table. Seasonal extremes and averages for various metals.**
