# Supplementary material for: Heavy metal distribution and ecological risk in surface sediments of the Bohai Sea
Source: PLoS One. 2025 Jun 27;20(6):e0326701. doi: 10.1371/journal.pone.0326701 (PMC12204586; doi:10.1371/journal.pone.0326701)
Supplement: S3 Table — (DOCX) [file pone.0326701.s003.docx]

| **Comprehensive pollution coefficient(C*_d_*)** | **Comprehensive potential ecological risk** |
| --- | --- |
| 2.69 | 41.82 |
| 3.04 | 33.91 |
| 1.94 | 18.59 |
| 1.91 | 21.63 |

**S3 Table. Comprehensive pollution of heavy metals in surface sediments in the central Bohai Sea.**
