# Supplementary material for: Heavy metal distribution and ecological risk in surface sediments of the Bohai Sea
Source: PLoS One. 2025 Jun 27;20(6):e0326701. doi: 10.1371/journal.pone.0326701 (PMC12204586; doi:10.1371/journal.pone.0326701)
Supplement: S4 Table — (DOCX) [file pone.0326701.s004.docx]

**S4 Table. Single factor results of heavy metal pollution in sediments in different seasons**

| **Month** | **Single factor pollution coefficient.(**$\boldsymbol{C}_{\boldsymbol{f}}^{\boldsymbol{i}}$**)** | | | | | |
| --- | --- | --- | --- | --- | --- | --- |
|  | **Cu** | **Zn** | **Pb** | **Cd** | **Hg** | **As** |
| May | 0.58 | 0.39 | 0.51 | 1.16 | 0.03 | 0.02 |
| Aug | 0.68 | 0.58 | 0.72 | 0.30 | 0.33 | 0.43 |
| October | 0.43 | 0.32 | 0.70 | 0.32 | 0.04 | 0.13 |
| December | 0.46 | 0.36 | 0.51 | 0.27 | 0.17 | 0.14 |
