# Supplementary material for: Heavy metal distribution and ecological risk in surface sediments of the Bohai Sea
Source: PLoS One. 2025 Jun 27;20(6):e0326701. doi: 10.1371/journal.pone.0326701 (PMC12204586; doi:10.1371/journal.pone.0326701)
Supplement: S5 Table — (DOCX) [file pone.0326701.s005.docx]

**S5 Table Potential ecological risk factors of heavy metals in surface sediments in different seasons.**

| **Month** | **Single factor ecological risk** | | | | | |
| --- | --- | --- | --- | --- | --- | --- |
|  | **Cu** | **Zn** | **Pb** | **Cd** | **Hg** | **As** |
| May | 2.91 | 0.39 | 2.572 | 34.78 | 1.01 | 0.15 |
| Aug | 3.38 | 0.58 | 3.617 | 8.87 | 13.12 | 4.34 |
| October | 2.16 | 0.32 | 3.500 | 9.65 | 1.67 | 1.29 |
| December | 2.29 | 0.36 | 2.573 | 8.12 | 6.89 | 1.39 |
