# Supplementary material for: Heavy metal distribution and ecological risk in surface sediments of the Bohai Sea
Source: PLoS One. 2025 Jun 27;20(6):e0326701. doi: 10.1371/journal.pone.0326701 (PMC12204586; doi:10.1371/journal.pone.0326701)
Supplement: S6 Table — (DOCX) [file pone.0326701.s006.docx]

**S6 Table Seasonal variations of heavy metals in sediments.**

| **Central Bohai Sea** | **Year** | **Cu**  **(mg/kg)** | **Zn**  **(mg/kg)** | **Pb**  **(mg/kg)** | **Cd**  **(mg/kg)** | **Hg (mg/kg)** | **As (mg/kg)** |
| --- | --- | --- | --- | --- | --- | --- | --- |
| Summer | 2011 | 16.84 | 41.87 | 11.36 | 1.65 | 0 | 0 |
| Winter | 2011 | 18.23 | 39.74 | 11.98 | 0.20 | 0 | 0 |
| Spring | 2013 | 17.49 | 31.29 | 12.86 | 0.58 | 0.005 | 0.230 |
| Summer | 2013 | 20.27 | 46.55 | 18.08 | 0.15 | 0.066 | 6.515 |
| Autumn | 2013 | 12.98 | 25.52 | 17.50 | 0.16 | 0.008 | 1.931 |
| Winter | 2013 | 13.76 | 28.42 | 12.87 | 0.14 | 0.034 | 2.088 |
| Summer | 2014 | 24.26 | 68.95 | 18.85 | 0.17 | 0 | 6.89 |
| Winter | 2015 | 27.61 | 68.7 | 23 | 0.14 | 0 | 5.73 |
| Summer | 2015 | 19.17 | 127.66 | 45.11 | 0.39 | 0 | 0 |
| Winter | 2016 | 21.81 | 55.73 | 21.23 | 0.16 | 0 | 9.21 |
| Summer | 2017 | 21 | 52.13 | 19.74 | 0.14 | 0 | 7.35 |
| Winter | 2018 | 25.56 | 63.49 | 21.21 | 0.15 | 0 | 6.58 |
| Summer | 2019 | 20.27 | 46.55 | 18.08 | 0.15 | 0.06 | 6.52 |
| Winter | 2019 | 13.76 | 28.42 | 12.87 | 0.14 | 0.03 | 2.08 |
| Summer | 2020 | 20.67 | 41.76 | 26.96 | 0.22 | 0.01 | 3.09 |
| Winter | 2020 | 11.20 | 38.43 | 11.68 | 0.18 | 0.01 | 1.91 |
| Summer | 2021 | 11.40 | 28.33 | 10.94 | 0.38 | 0.01 | 10.72 |
| Winter | 2021 | 12.09 | 41.01 | 28.19 | 0.27 | 0.01 | 2.12 |
