# Supplementary material for: Heavy metal distribution and ecological risk in surface sediments of the Bohai Sea
Source: PLoS One. 2025 Jun 27;20(6):e0326701. doi: 10.1371/journal.pone.0326701 (PMC12204586; doi:10.1371/journal.pone.0326701)
Supplement: S7 Table — (DOCX) [file pone.0326701.s007.docx]

**S7 Table. Annual Variations in Heavy Metal Accumulation in Sediments.**

| **Year** | **Cu**  **(mg/kg)** | **Zn**  **(mg/kg)** | **Pb**  **(mg/kg)** | **Cd**  **(mg/kg)** | **Hg (mg/kg)** | **As (mg/kg)** |
| --- | --- | --- | --- | --- | --- | --- |
| 2011 | 35.07 | 81.61 | 23.34 | 1.85 | 0.00 | 0.00 |
| 2011 | 64.50 | 131.78 | 61.31 | 1.03 | 0.11 | 10.76 |
| 2013 | 24.26 | 68.95 | 18.85 | 0.17 | 0.00 | 6.89 |
| 2013 | 46.78 | 196.36 | 68.11 | 0.53 | 0.00 | 5.73 |
| 2013 | 21.81 | 55.73 | 21.23 | 0.16 | 0.00 | 9.21 |
| 2013 | 21.00 | 52.13 | 19.74 | 0.14 | 0.00 | 7.35 |
| 2014 | 25.56 | 63.49 | 21.21 | 0.15 | 0.00 | 6.58 |
| 2015 | 34.04 | 74.97 | 30.95 | 0.29 | 0.09 | 8.60 |
| 2015 | 31.87 | 80.19 | 38.64 | 0.40 | 0.02 | 5.00 |
| 2016 | 23.49 | 69.34 | 39.13 | 0.65 | 0.02 | 12.84 |
| 2017 | 35.07 | 81.61 | 23.34 | 1.85 | 0.00 | 0.00 |
| 2018 | 64.50 | 131.78 | 61.31 | 1.03 | 0.11 | 10.76 |
| 2019 | 24.26 | 68.95 | 18.85 | 0.17 | 0.00 | 6.89 |
| 2019 | 46.78 | 196.36 | 68.11 | 0.53 | 0.00 | 5.73 |
| 2020 | 21.81 | 55.73 | 21.23 | 0.16 | 0.00 | 9.21 |
| 2020 | 21.00 | 52.13 | 19.74 | 0.14 | 0.00 | 7.35 |
| 2021 | 25.56 | 63.49 | 21.21 | 0.15 | 0.00 | 6.58 |
| 2021 | 34.04 | 74.97 | 30.95 | 0.29 | 0.09 | 8.60 |
