# Supplementary material for: Heavy metal distribution and ecological risk in surface sediments of the Bohai Sea
Source: PLoS One. 2025 Jun 27;20(6):e0326701. doi: 10.1371/journal.pone.0326701 (PMC12204586; doi:10.1371/journal.pone.0326701)
Supplement: Attachment — Submitted filename: Reviewed Manuscript_PONE-D-25-01587.docx [file pone.0326701.s008.docx]

**Title: Distribution Characteristics and Pollution Assessment of Heavy Metals in Surface Sediments in the Central Region of the Bohai Sea**

- **Comment**: The title is informative but could be more concise for clarity and impact. Consider removing redundant terms like "Distribution Characteristics and Pollution Assessment."
- **Recommendation**: Revise to something like "Heavy Metal Distribution and Ecological Risk in Surface Sediments of the Bohai Sea."

**Abstract**

- **Comment**: The abstract is comprehensive but overly detailed, especially with numerical values. This can overwhelm the reader.
- **Recommendation**: Summarize key findings concisely, focusing on trends, risks, and implications for environmental management.

**Introduction**

- **Strengths**: Provides a strong context, linking heavy metal pollution with ecological risks and human activities in the Bohai Sea.
- **Weaknesses**: The introduction is slightly repetitive (e.g., overlapping details about river inputs and sediment roles).
- **Recommendation**: Streamline by focusing on the knowledge gap and objectives. Move some background information to the discussion.

**Methods**

**Study Area and Sample Collection**

- **Comment**: The study area description is detailed but lacks clear justification for choosing the central Bohai Sea.
- **Recommendation**: Explicitly state why this region was selected and its importance compared to adjacent areas.

**Quality Assurance**

- **Strengths**: The QA/QC measures are thorough.
- **Recommendation**: Include references for the detection methods (e.g., GB standards) for better credibility.

**Results**

**Heavy Metal Content**

- **Comment**: Tables and figures are detailed but could be overwhelming due to excess numerical values.
- **Recommendation**: Highlight critical trends (e.g., exceedance rates, highest/lowest concentrations) in the text rather than listing all values.

**Seasonal Variations**

- **Comment**: Seasonal trends are well-documented, but the discussion of influencing factors could be deeper.
- **Recommendation**: Discuss how these variations align with known hydrodynamic and anthropogenic processes in the Bohai Sea.

**Discussion**

**Seasonal Variation**

- **Strengths**: Comprehensive comparisons with past studies.
- **Weaknesses**: Too many references to specific concentrations; the focus on ecological implications is limited.
- **Recommendation**: Emphasize the ecological and management implications of seasonal changes instead of re-analyzing the numbers.

**Ecological Risks**

- **Comment**: The risk assessment is solid, but the methodology explanation could be simplified.
- **Recommendation**: Reduce redundancy in explaining the risk indices and focus on the implications for sediment management.

**Long-term Trends**

- **Strengths**: Excellent temporal comparison with historical data.
- **Weaknesses**: The explanation of interannual fluctuations could be more robust.
- **Recommendation**: Link temporal trends to regional policy changes, industrial activities, or specific events like oil spills.

**Conclusion**

- **Comment**: The conclusion is repetitive and lacks actionable insights.
- **Recommendation**: Summarize the key findings in one paragraph and add specific recommendations for policymakers and future research.

**Tables and Figures**

- **Comment**: Figures are informative but could benefit from improved design (e.g., larger fonts, consistent color schemes).
- **Recommendation**: Ensure all figures are publication-ready with clear legends and high resolution.

**References**

- **Comment**: The references are recent and relevant but lack diversity in geographical scope.
- **Recommendation**: Include more global studies on heavy metal pollution for broader context.
