# Supplementary material for: Heavy metal distribution and ecological risk in surface sediments of the Bohai Sea
Source: PLoS One. 2025 Jun 27;20(6):e0326701. doi: 10.1371/journal.pone.0326701 (PMC12204586; doi:10.1371/journal.pone.0326701)
Supplement: Attachment — Submitted filename: PLOS review 25-01587.docx [file pone.0326701.s009.docx]

- The Manuscript assesses surface sediment contamination in Bohai sea region. The authors has put in good efforts for such extensive sampling.
- As the area is highly researched already by several researchers and hence, the present work do not present a novel or knowledge gap fulfilment. Line 105-108: There is already much research done on heavy metal conc. In Bohai sea. Already many of them included in literature.
- The sediment samples collection is already a decade old. Also, is the present analysis using AAS is done presently or during 2013 which needs to be made clear.
- Line 125 – capitalize
- As the overall research is carried out on the base of 2013 year sampling. I think the data is a more than a decade old and to put forward it in present scenario will have less importance in present context.
- Moreover, already good quantity of work is already been undertaken in the study area hence the study does not fill the knowledge gap.
- As already mentioned in introduction and discussion section, the study area is a highly polluted area, which the result presented the moderate to low quantity of pollution.
- The seasonal changes in the heavy metal concentration are not properly justifiable. Specially, the study mentions a lot anthropogenic activities in the bay region. Although, the sources and activities are not well discussed.
- I think, the authors can revise the MS and modify the data to form a comprehensive review with all anthropogenic activities.

Xu, X., Cao, Z., Zhang, Z., Li, R., & Hu, B. (2016). Spatial distribution and 715

pollution assessment of heavy metals in the surface sediments of the Bohai and 716

Yellow Seas.

Wang, X., Fu, R., Li, H., Zhang, Y., Lu, M., Xiao, K., ... & Xiong, Y. (2020). Heavy 720

metal contamination in surface sediments: A comprehensive, large-scale 721

evaluation for the Bohai Sea, China. *Environmental Pollution*, *260*, 113986.

Ding, X., Ye, S., Laws, E. A., Mozdzer, T. J., Yuan, H., Zhao, G., ... & Wang, J. 710

(2019). The concentration distribution and pollution assessment of heavy metals 711

in surface sediments of the Bohai Bay, China. *Marine pollution bulletin*, *149*,

Liu, X., Jiang, X., Liu, Q., Teng, A., & Xu, W. (2016). Distribution and pollution 662

assessment of heavy metals in surface sediments in the central Bohai Sea, China: 663

a case study. *Environmental earth sciences*, *75*, 1-14.

Zhu, A., Liu, J., Qiao, S., & Zhang, H. (2020). Distribution and assessment of

heavy metals in surface sediments from the Bohai Sea of China. *Marine Pollution*

*Bulletin*, 153, 110901. https://doi.org/10.1016/j.marpolbul.2020.110901
